# The contribution of astrocytes to the regulation of cerebral blood flow

**DOI:** 10.3389/fnins.2014.00103

**Published:** 2014-05-09

**Authors:** Clare Howarth

**Affiliations:** Department of Psychology, University of SheffieldSheffield, UK

**Keywords:** astrocyte, neurovascular coupling, cerebral blood flow, calcium, functional hyperemia

## Abstract

In order to maintain normal brain function, it is critical that cerebral blood flow (CBF) is matched to neuronal metabolic needs. Accordingly, blood flow is increased to areas where neurons are more active (a response termed functional hyperemia). The tight relationships between neuronal activation, glial cell activity, cerebral energy metabolism, and the cerebral vasculature, known as neurometabolic and neurovascular coupling, underpin functional MRI (fMRI) signals but are incompletely understood. As functional imaging techniques, particularly BOLD fMRI, become more widely used, their utility hinges on our ability to accurately and reliably interpret the findings. A growing body of data demonstrates that astrocytes can serve as a “bridge,” relaying information on the level of neural activity to blood vessels in order to coordinate oxygen and glucose delivery with the energy demands of the tissue. It is widely assumed that calcium-dependent release of vasoactive substances by astrocytes results in arteriole dilation and the increased blood flow which accompanies neuronal activity. However, the signaling molecules responsible for this communication between astrocytes and blood vessels are yet to be definitively confirmed. Indeed, there is controversy over whether activity-induced changes in astrocyte calcium are widespread and fast enough to elicit such functional hyperemia responses. In this review, I will summarize the evidence which has convincingly demonstrated that astrocytes are able to modify the diameter of cerebral arterioles. I will discuss the prevalence, presence, and timing of stimulus-induced astrocyte calcium transients and describe the evidence for and against the role of calcium-dependent formation and release of vasoactive substances by astrocytes. I will also review alternative mechanisms of astrocyte-evoked changes in arteriole diameter and consider the questions which remain to be answered in this exciting area of research.

## Introduction

For normal functioning of the brain to be maintained it is critical that increases in neuronal energy demands are met by changes in local blood flow with high temporal and spatial resolution. This necessitates close connections between neurons, glia, and the energy metabolism and blood supply of the brain. Increased neuronal activity is accompanied by an increase in local cerebral blood flow (CBF), a phenomenon termed functional hyperemia. It is this increase in CBF and oxygenation which underlies BOLD functional MRI (fMRI). BOLD fMRI is commonly used as a surrogate measure of neural activity. A valid interpretation of such data requires a thorough understanding of the cellular basis of the BOLD signal. While a coupling between cerebral energy consumption and neuronal activity was originally suggested over a century ago (Roy and Sherrington, 1890), the exact relationship remains an active area of research. Although neuronal activity induced increases in blood flow are due, at least in part, to the direct action of neurons [via glutamate-evoked release of nitric oxide (NO)] on arteriole smooth muscle (Fergus and Lee, 1997), over the past decade there has been extensive research (Zonta et al., 2003; Mulligan and MacVicar, 2004; Filosa et al., 2006; Takano et al., 2006) determining the role which astrocytes, and activity-induced Ca^2+^ signals within astrocytes, may play (as discussed in recent reviews by Attwell et al., 2010; Petzold and Murthy, 2011).

**Figure 1:**
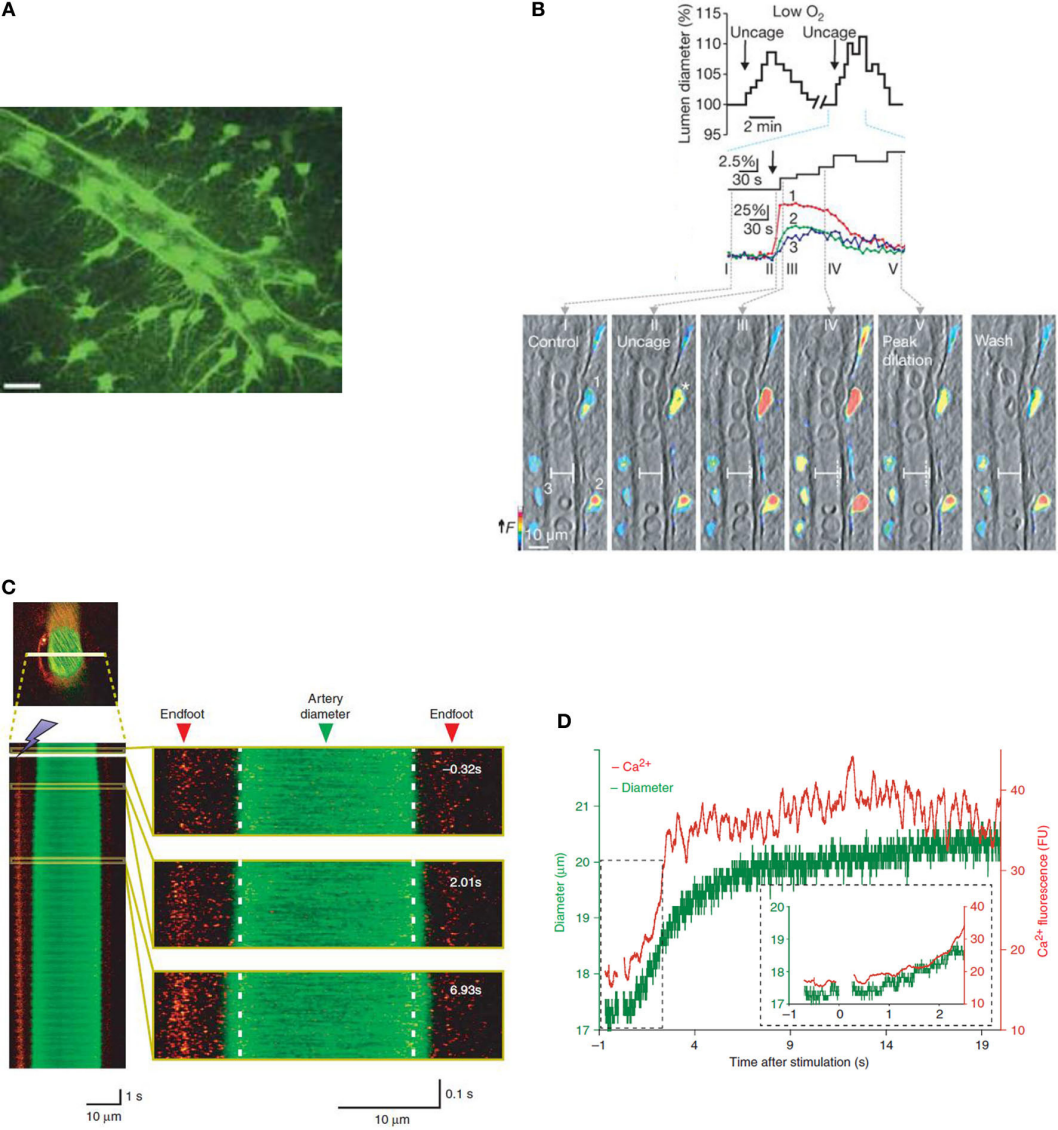
**Increases in astrocyte [Ca^2+^]_i_ are accompanied by changes in vessel diameter *in vitro* and *in vivo*. (A)** Two-dimensional projection of two photon microscopy images showing that GFP-positive astrocytes and their endfeet delineate an arteriole. Scale bar is 20 μm. Reprinted by permission from Macmillan Publishers Ltd., Nature (Mulligan and MacVicar, 2004) copyright (2004). **(B)** Astrocytes in brain slice from rat loaded with calcium indicator dye (rhod-2/AM) and caged calcium compound (DMNPE-4/AM). Uncaging calcium within astrocytes causes an increase in [Ca^2+^]_i_ in astrocyte soma and endfeet which preceded vasodilation (top). Vessel and pseudo-colored endfoot Ca^2+^ changes correspond to times in top image. Reprinted by permission from Macmillan Publishers Ltd., Nature (Gordon et al., 2008) copyright (2008). **(C)** Vessel diameter changes can be measured in mouse cortex *in vivo* using 2-photon microscopy in line scan mode. Here, calcium is measured using rhod-2/AM and vessels are visualized with a dextran-coupled dye. Left, line scan image of an artery exposed to photolysis of caged Ca^2+^ which increases astrocyte [Ca^2+^]_i_. Astrocytic Ca^2+^ and vessel diameter increase almost simultaneously following photolysis. Right, larger views of line scan section indicated in yellow boxes. **(D)** Time course of changes in astrocyte [Ca^2+^]_i_ and vessel diameter in **(C)**. Reprinted by permission from Macmillan Publishers Ltd., Nature Neuroscience (Takano et al., 2006) copyright (2006).

Being situated in the synaptic cleft and having multiple endfeet which are opposed to smooth muscle cells (Figure 1A), astrocytes can act as a “bridge,” relaying information about changes in synaptic activity between neurons and the vasculature, ensuring that neuronal energy demands are met.

## Initial *in vitro* evidence demonstrated that astrocytes can regulate arteriole diameter

Initial studies revealing a potential role of astrocytes in neurovascular coupling were performed *in vitro* using acute brain slices and whole mount retina. This *in vitro* research has resulted in convincing evidence that astrocytes are able to control vascular diameter (Figure 1B). During neuronal activity, glutamate is released and acts via neuronal NMDA receptors to activate neuronal nitric oxide synthase (nNOS), resulting in the release of NO. NO acts on smooth muscle cells, increasing blood flow via a cGMP pathway (Fergus and Lee, 1997). However, in addition to triggering neuronal NO-evoked effects on the vasculature, neuronally released glutamate can act on astrocyte metabotropic glutamate receptors (mGluR), raising astrocyte [Ca^2+^]_i_ (Zonta et al., 2003; Takano et al., 2006). Over a decade ago, observations of astrocyte soma and endfeet [Ca^2+^]_i_ signals which were well-timed with vessel diameter changes in response to mGluR activation were the first evidence that astrocytes may contribute to neurovascular coupling (Zonta et al., 2003). This work implicated cyclooxygenase enzymes (COX) in the downstream signaling pathway leading from increased astrocyte [Ca^2+^]_i_ to vessel dilation. An increase in astrocytic [Ca^2+^]_i_ can result in the production of arachidonic acid (AA) via phospholipase A2 (PLA2), a Ca^2+^ sensitive enzyme highly expressed in astrocytes (Farooqui et al., 1997; Cahoy et al., 2008). AA is subsequently metabolized to COX and cytochrome P450 epoxygenase derivatives [prostaglandin E_2_ (PgE_2_) and epoxyeicosatrienoic acids (EETs), respectively]. These vasoactive metabolites can be released from the astrocyte endfeet, apposed to arterioles, resulting in activation of smooth muscle K^+^ channels and vasodilation (although see Dabertrand et al. (2013) who suggest that PgE_2_ may constrict, rather than dilate, isolated parenchymal arterioles).

**Figure 2:**
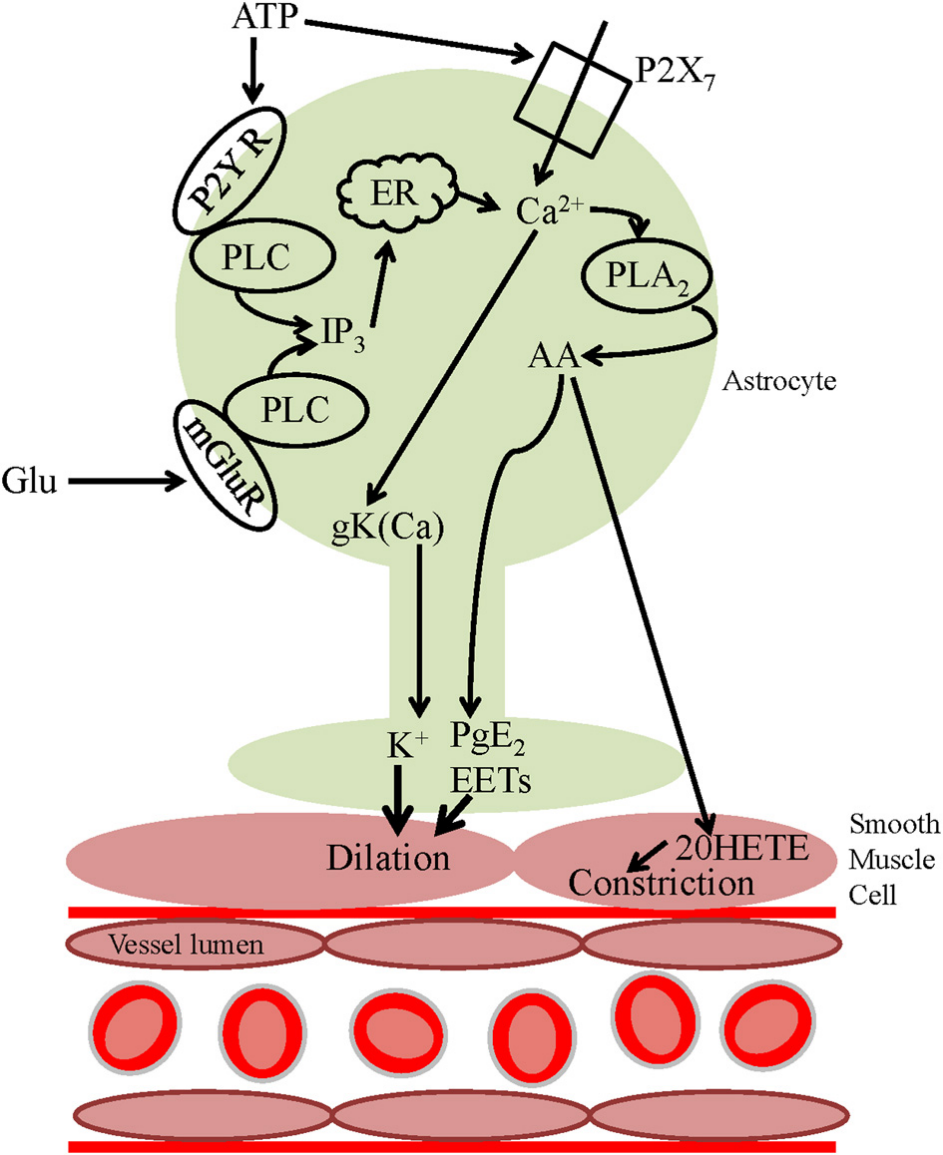
**Astrocyte calcium-dependent vasoactive signaling pathways**. Neuronally released glutamate can act on astrocyte mGluRs, activating PLC, and increasing astrocyte [Ca^2+^]_i_, activating PLA2 resulting in the release of AA from the plasma membrane. AA can be metabolized within the astrocyte to form PgE_2_ or EETs which are released and act on smooth muscle cells, evoking vasodilation. Alternatively, AA can be released and act on smooth muscle cells where it is metabolized to the vasoconstrictor 20-HETE. ATP can activate Ca^2+^-mediated downstream vasoactive pathways either by acting on P2Y receptors and activating PLC or via P2X_7_ receptors, increasing [Ca^2+^]_i_. An alternative vasoactive pathway downstream of the [Ca^2+^]_i_ increase is the activation of BK_Ca_ channels and subsequent efflux of the vasodilator K^+^.

In addition to AA being metabolized within the astrocyte, it can diffuse to arteriole smooth muscle, producing the vasoconstrictor 20-HETE via ω-hydroxylases (Roman, 2002). Shortly after the demonstration that astrocyte [Ca^2+^]_i_ increases were closely linked to vasodilations, two photon photolysis of caged calcium directly within the somata of astrocytes was used to trigger a [Ca^2+^]_i_ transient within the astrocyte and evoked vasoconstriction (Mulligan and MacVicar, 2004). Pharmacology experiments revealed the importance of PLA2 and it was proposed that 20-HETE, a vasoconstrictor, was generated from AA, which was formed in the astrocytes. 20-HETE inhibits smooth muscle K^+^ conductances to depolarize and contract smooth muscle cells (Lange et al., 1997). Thus, astrocyte [Ca^2+^]_i_ entry can trigger either vasodilation (Zonta et al., 2003; Filosa et al., 2004) or vasoconstriction (Mulligan and MacVicar, 2004) depending on which signaling pathway dominates (Figure 2).

The retina is an ideal system in which to study blood flow regulation in response to local signals as its low density of blood vessels requires the ability to efficiently match the local blood supply to local neuronal metabolic needs (Funk, 1997). The observation that glial [Ca^2+^]_i_ transients were closely correlated in time with changes in arteriole diameter was extended to the case of the retina where both vasodilations and constrictions were reported to be evoked by either physiological light stimulation or uncaging of Ca^2+^ in Muller cells (Metea and Newman, 2006). In agreement with the previous findings in hippocampal slices (Mulligan and MacVicar, 2004), 20-HETE was implicated as the vasoconstrictor molecule in the retina. However, in contrast to findings in cortical slices (Zonta et al., 2003), the data suggested that conversion of AA to EETs, rather than to PgE_2_, caused arteriole dilations in the retina. The hunt was on to find the variable which selects a dilatory response over a constrictive one and vice versa.

While *in vitro* studies have several advantages, including the ability to control various cellular elements, there are technical limitations to this approach which are worth noting. A lack of myogenic tone, due to a lack of perfusion and intraluminal pressure (Iadecola and Nedergaard, 2007), can result in vessels being maximally dilated. To compensate for this loss of tone, in many studies, slices are pre-treated with a vasoconstrictor (Zonta et al., 2003; Filosa et al., 2004; Metea and Newman, 2006). However, preconstriction has been shown to alter the direction of arteriolar responses (Mulligan and MacVicar, 2004). Furthermore, many experiments are carried out at non-physiological temperatures, e.g., with brain slices maintained at room temperature (Mulligan and MacVicar, 2004; Gordon et al., 2008).

## How is the direction of arteriole diameter change determined?

NO, which can bind to the heme moiety and inactivate cytochrome P450 enzymes (Fleming, 2001; Roman, 2002), was suggested to determine the direction of retinal arteriole diameter change (Metea and Newman, 2006). While in the brain neural activity and the resulting NO production has been shown to correspond to increases in blood flow (Akgoren et al., 1994), in the retina the occurrence of vasoconstrictions dominated as NO levels increased (Metea and Newman, 2006). This finding was in agreement with pharmacological inhibition of NO synthase, which converted astrocyte-evoked vasoconstrictions to vasodilations in brain slices (Mulligan and MacVicar, 2004). A possible explanation for this observation is that preconstriction of vessels by L-NAME, which was used to inhibit NO synthase, increases the basal tone of vessels and, hence, may predispose them to dilate to other factors (Blanco et al., 2008). Many of the enzymes suggested to be responsible for signaling downstream of the increase of astrocyte [Ca^2+^]_i_ are sensitive to NO (e.g., CYP4A which produces 20-HETE) (Fleming, 2001; Roman, 2002) suggesting that a complex relationship may exist between NO levels and neurovascular coupling signaling pathways. Differing basal NO levels may exist in different preparations, hence pathways may be inhibited to varying degrees. This may explain why some groups reported only constrictions (Mulligan and MacVicar, 2004) while others reported constrictions and dilations (Metea and Newman, 2006).

Metabolic factors, such as partial pressure of oxygen (pO_2_) (Offenhauser et al., 2005) and the extracellular lactate concentration (Hu and Wilson, 1997) change rapidly within the parenchyma during neural activity. Gordon et al. (2008) performed experiments in acute brain slices and proposed that such metabolic factors may play a role in determining the direction of arteriole diameter changes. The level of oxygen present in the aCSF (artificial CSF) used in these experiments was found to determine the direction of arteriole diameter change in response to uncaging calcium within the soma of astrocytes (Gordon et al., 2008). At higher levels of O_2_ (aCSF bubbled with 95% O_2_ and 5% CO_2_, typical of acute brain slice experiments), vasoconstrictions were triggered, while at lower O_2_ levels vasodilations dominated (Figure 1B). The lower O_2_ level (aCSF bubbled with 20% O_2_), resulted in a pO_2_ which mimics the lower end of physiological measurements *in vivo* (Offenhauser et al., 2005). At the lower oxygen levels used, both lactate and adenosine levels were increased compared to under conditions of higher O_2_ and vasodilation was proposed to be dominant due to two mechanisms. Firstly, as uptake of PgE_2_ by the prostaglandin transporter is inhibited by extracellular lactate (Chan et al., 2002), there is an accumulation of extracellular PgE_2_ following [Ca^2+^]_i_-evoked PgE_2_ release by astrocytes, thus facilitating the vasodilatory response. Secondly, the increased levels of adenosine were proposed to be acting on A2A receptors on the smooth muscle itself, blocking Ca^2+^ channels (Murphy et al., 2003) and preventing vasoconstriction. In agreement with these findings, in *ex vivo* retina, the incidence of light-evoked vasoconstrictions was lower in 21% O_2_ compared to 100% O_2_. Additionally, at the lower oxygen level, a PgE_2_ component of vasodilation became salient (Mishra et al., 2011). Whether such a mechanism plays a functional role *in vivo* remains to be proven. Although changing tissue pO_2_ by breathing high or low oxygen has been shown to change basal CBF and arteriole diameter in the direction predicted by *in vitro* experiments (McCalden et al., 1984; Mishra et al., 2011), hyperoxia had no effect on light-evoked dilations or flow in the retina *in vivo* (Mishra et al., 2011). Furthermore, an increased tissue pO_2_ failed to alter the functional hyperemia response to sensory stimulation (Lindauer et al., 2010). Lin et al. (2010) recently published human NMR spectroscopy studies showing that CBF increases were positively correlated with lactate production while being negatively correlated with the percentage change in oxygen consumption (CMRO_2_). These findings suggest that task-induced CBF responses are mediated by factors other than the demand for oxygen. In order to test the *in vivo* relevance of the findings of Gordon et al. (2008), it may be more appropriate to test the end effectors predicted by their experiments, i.e., lactate and adenosine.

## Alternative mechanisms of astrocyte control of CBF

In addition to the mGluR-evoked mechanisms of CBF regulation, there is evidence for a further glutamate-dependent pathway. In the olfactory bulb, intrinsic optical signal (IOS) changes (used as a proxy for CBF measurements) in response to odor stimulation were found to be unaffected by blocking AMPA/NMDA receptors nor mGluRs (Gurden et al., 2006). However, the increase in CBF was reduced when glial glutamate transporters were blocked. This work was expanded by Schummers et al. (2008) who demonstrated that, in visual cortex, the astrocytic [Ca^2+^]_i_ signal and the change in IOS in response to a visual stimulus were significantly reduced when glial glutamate transporters were blocked. Furthermore, blocking glial glutamate transporters reduced odor-evoked increases in both erythrocyte velocity and flux in the olfactory bulb [even after controlling for potentially higher receptor activity after transporter blockade Petzold et al. (2008)]. In contrast to experiments in the visual cortex (Schummers et al., 2008) however, Petzold et al. (2008) observed no significant change of the calcium response in astrocyte somata when blocking glial glutamate uptake. While further experimentation is needed to resolve the signaling molecules which underlie this mechanism of CBF control, these data suggest that calcium-independent vasodilatory pathways may exist. Indeed, IP_3_-independent stimulation-induced vasodilation has recently been observed in the cortex of IP_3_ knockout mice (Nizar et al., 2013). The role of astrocyte Ca^2+^ signaling in the regulation of CBF is currently hotly debated and will be discussed later in this review.

In contrast to brain slices, glutamate is largely ineffective in evoking glial [Ca^2+^]_i_ increases in the retina. In retina, neuron-to-glia signaling, and resulting vasoactivity, is mediated by neuronal release of ATP and activation of purinergic P2Y receptors (Newman, 2005; Metea and Newman, 2006). Activation of P2Y receptors (which are highly expressed in astrocyte endfeet: Simard et al., 2003), activates phospholipase C (PLC) and the downstream calcium-dependent signaling pathways discussed above (Figure 2). ATP can also act on glial P2X_7_ receptors, resulting in an increase in astrocyte [Ca^2+^]_i_ (Carrasquero et al., 2009; Habbas et al., 2011) and triggering the formation and release of vasoactive substances (Figure 2). In addition to neuronally released ATP, calcium-dependent ATP exocytosis by glial cells may occur (Pangrsic et al., 2007; Blum et al., 2008). ATP which is released into the extracellular space is rapidly hydrolyzed to form adenosine (Xu and Pelligrino, 2007) which has been shown to be vasodilatory in both the cerebral cortex and cerebellum, and is thought to be involved in functional hyperemia *in vivo* (Dirnagl et al., 1994; Akgoren et al., 1997; Shi et al., 2008).

Increases in extracellular concentrations of K^+^ cause vasodilation in cerebral arterioles (Kuschinsky and Wahl, 1978). Although the original hypothesis of “astrocyte K^+^ siphoning” (Paulson and Newman, 1987) has been disproved (Metea et al., 2007), a calcium-dependent mechanism by which astrocytes may contribute to the regulation of CBF via K^+^ has been demonstrated (Filosa et al., 2006). BK_Ca_ channels in astrocyte endfeet were shown to be activated following neuronal activity-evoked increases in astrocytic [Ca^2+^]_i_ via mGluR activation. The resulting local increase in extracellular K^+^ activated K_ir_ channels (K_ir_2.1) on the smooth muscle cell, hyperpolarizing the cell and leading to vasodilation. This work is consistent with *in vivo* studies inhibiting BK_Ca_ channels (Gerrits et al., 2002) and K_ir_ channels (Leithner et al., 2010), both of which were found to result in an attenuation of the CBF increase evoked by somatosensory activation. However, as glial membrane potentials are close to the equilibrium potential for K^+^ (Kuffler et al., 1966), increasing K^+^ conductance may not result in an increased net efflux of K^+^. Furthermore, as the contribution of endfeet K^+^ efflux (via glial K_ir_4.1 channels) has been disproved in the retina (Metea et al., 2007), its role in the cortex needs to be verified.

## Do astrocytes play a role in the regulation of CBF *in vivo*?

Several experimental models have been used to investigate the role of astrocytes in the regulation of CBF *in vivo* including: uncaging of Ca^2+^ within astrocytes, somatosensory stimulation, pharmacological inhibition, and genetic deletion.

When Ca^2+^ was uncaged within astrocyte endfeet, triggering an increase in astrocyte [Ca^2+^]_i_, dilation of an adjacent arteriole was observed (Figures 1C,D) (Takano et al., 2006). In agreement with the suggestion that AA conversion to PgE_2_ underlies the dilation, inhibition of COX-1 but not COX-2 enzymes blocked the vasodilations. However, controversy remains regarding the role of COX-1 in neural activity-evoked vasodilation. While COX-1 inhibition (with a high dose of SC560) can inhibit the CBF response to odorant stimulation in the olfactory bulb (Petzold et al., 2008) or uncaging of Ca^2+^ in astrocytes in the cortex (Takano et al., 2006), lower doses of SC560 have no effect on the CBF response to whisker stimulation (Niwa et al., 2001; Lecrux et al., 2011; Liu et al., 2012). Furthermore, genetic deletion of COX-1 had no effect on functional hyperemia (Niwa et al., 2001). In contrast, pharmacological inhibition or genetic knockout of COX-2 attenuates the CBF response to neuronal activation (Niwa et al., 2000). As COX-2 is more highly expressed in neurons than astrocytes, these data have led to the suggestion that neuronal COX activity may underlie the component of functional hyperemia which is mediated by COX products. Recent data suggests that photolysis of caged Ca^2+^ might artifactually produce vasodilation via glutamate-permeable anion channels. Activation of these channels (either by calcium or astrocytic volume changes following photolysis) leads to glutamate release and an mGluR-mediated increase in mEPSC frequency. Photolysis-induced astrocytic glutamate release activates neuronal mGluRs and NMDA/AMPA receptors resulting in K^+^ efflux and neuronal depolarization (and potentially smooth muscle cell hyperpolarization due to increased extracellular K^+^) (Wang et al., 2013). This effect may explain the differing effects of COX-1 inhibition on sensory stimulus-mediated vasodilation vs. photolysis-mediated vasodilation. In addition, regional heterogeneity of COX-1 expression (as has been found for nNOS) may offer a further explanation for the differing effects of COX-1 inhibition which have been observed.

Although some groups have used sensory stimuli to investigate the signaling pathways underlying astrocyte-mediated CBF changes (e.g., Zonta et al., 2003; Petzold et al., 2008), much of the evidence for astrocytic mGluR-mediated vasodilations is based on *in vitro* work using tissue from juvenile rodents (e.g., Zonta et al., 2003; Mulligan and MacVicar, 2004). A role for mGluR-mediated vasodilations in adult rodents remains contentious. Recent research has suggested that expression levels of mGluR5 alter with development, being undetectable beyond postnatal week 3 (Sun et al., 2013). In agreement with this finding, Calcinaghi et al. (2011), using a highly specific mGluR5 blocker, found no evidence for a role of mGluR5 in the onset or maintenance of CBF increases in the whisker barrel of adult anesthetized rats in response to brief whisker stimulation. Furthermore, blockade of mGluRs in the olfactory bulb had no effect on the hemodynamic response to odor stimulation (Gurden et al., 2006). However, in contradiction to these results, mGluR5-antagonist sensitive sensory simulation-evoked astrocyte [Ca^2+^]_i_ transients in the barrel cortex of adult mice have been reported (Wang et al., 2006; Lind et al., 2013). In the olfactory bulb, Petzold et al. (2008) reported that the mGluR5 antagonist, MPEP, decreased vasodilations, supporting the idea that functional hyperemia is mediated, at least in part, by mGluR5, which, within the glomerular layer is expressed exclusively in astrocytes. Vasodilations were also reduced by inhibiting COX-1, suggesting that the functional hyperemia mediated by astrocytic mGluR5 depends on COX-1 activity. It remains unclear, therefore, under what conditions mGluR5 plays a role in neurovascular coupling.

Several additional factors may explain the discrepencies observed in different studies. Regional differences in expression of mGluR5 and/or the importance of mGluR-mediated signaling for the regulation of CBF may exist (MPEP reduces fMRI responses to hindpaw stimulation in rat primary cortex by only 18%, compared to 66% in striatum: Sloan et al., 2010). mGluR5 may be upregulated in reactive astrocytes (Aronica et al., 2000), suggesting that the role of astrocytic mGluR5 in neurovascular coupling may be associated more with non-physiological conditions. The recruitment of astrocyte calcium-mediated vasodilation may depend upon the frequency of stimulation used. Wang et al. (2006) demonstrated that astrocyte calcium signals in the barrel cortex of mice were a function of frequency, with signals rarely evoked by a 1 Hz whisker stimulation and peaking in response to 5Hz stimulation (although this may only occur in the anesthetized state, see Thrane et al., 2012). Furthermore, recent imaging of neuronal and astrocytic calcium signals in the rat somatosensory cortex has shown that high frequency activation of the forepaw (a 10 Hz but not a 1 Hz stimulus) leads to a late component of vasodilation that is correlated with increased astrocyte calcium and increased CBF as measured by fMRI BOLD signals (Schulz et al., 2012). The findings discussed throughout this review suggest that there is a complex interaction of many factors (both astrocytic and neuronal) determining how CBF is controlled, both basally and in response to neural activity. The task of studying the cellular functionality of astrocytes and/or neurons is thus a challenging one.

In addition to the vasodilations described above, there is *in vivo* evidence for astrocyte [Ca^2+^]_i_ transients resulting in vasoconstriction. Two-photon imaging of astrocytes bulk loaded with calcium indicator dyes revealed that vasoconstrictions of penetrating cortical arterioles occurred during spreading depression (SD) at the onset of the fast astrocytic Ca^2+^ wave (Chuquet et al., 2007). Inhibiting either PLA2 or the refilling of internal calcium stores reduced the SD-induced vasoconstriction, suggesting that astrocytes mediate SD-induced vasoconstrictions via PLA2-mediated AA release.

In summary, the evidence suggests that in response to neural activity, astrocyte [Ca^2+^]_i_ increases and vasoactive messengers are released from astrocytic endfeet. Thus, astrocytes may evoke changes in arteriole diameter and regulate CBF.

## Are activity-evoked astrocyte calcium transients widespread and fast enough to contribute to neurovascular coupling?

Although a large body of evidence has been acquired over the past decade suggesting that astrocytes are potential mediators of functional hyperemia, the idea remains controversial. The presence, prevalence, and timing of astrocyte Ca^2+^ signaling in response to neural activity and its role in the regulation of CBF is currently hotly debated. In a recent review, Cauli and Hamel (2010) discuss the relative timings of astrocytic and neuronal calcium responses to neuronal activity. Rapid calcium events are thought to reflect activation of ionotropic receptors (which are expressed frequently in neurons), while slower calcium responses are proposed to reflect activation of metabotropic receptors (expressed by astrocytes and neurons) and the release of calcium from intracellular stores. These calcium signal dynamics agree with the observation that calcium events in neurons often precede those in astrocytes (Wang et al., 2006; Schummers et al., 2008; Nizar et al., 2013). These data would suggest that astrocytes may only contribute to functional hyperemia in the late phase of the response. Recent studies have suggested that arteriole dilations resulting from neural activity may not only precede astrocytic [Ca^2+^]_i_ signals (Nizar et al., 2013) but can, in fact, occur in the absence of glial [Ca^2+^]_i_ signals (Schulz et al., 2012).

Using *in vivo* 2-photon imaging of astrocytes, Wang et al. (2006) reported whisker stimulation-evoked astrocyte [Ca^2+^]_i_ transients in the barrel cortex which peak several seconds post stimulation. Such transients are too slow to trigger the hemodynamic response to neural activity, which occurs anywhere from a few hundred milliseconds to a couple of seconds after the onset of neuronal activity (Kleinfeld et al., 1998; Devor et al., 2003; Zonta et al., 2003). This idea is supported by evidence suggesting that there is a long lag time between the onset of stimulation and astrocyte [Ca^2+^]_i_ transients (Schulz et al., 2012; Thrane et al., 2012) and that, following forepaw stimulation, the onset of astrocyte calcium responses may lag behind the onset of arteriole dilation at the same depth within the cortex (Nizar et al., 2013). In this last study (as is common in such studies), bulk loading of the calcium indicator dye, Oregon Green Bapta-1 (OGB-1) was used to measure calcium signals in both neurons and astrocytes. During data analysis the astrocyte region of interest (ROI) was minimized in order to avoid contamination from neuropil signals, which were suggested to account for the initial rapid calcium transients sometimes observed within an astrocyte ROI. Such rapid transients were not observed in astrocytes when using the calcium indicator dye Fluo-4, which was absent in neurons. The difficulty in determining with 100% certainty whether a calcium signal is within an astrocyte, astrocytic process, or neuropil highlights the need for the development both of improved sensitivity of 2-photon detection and of better dye localization. However, other studies also using *in vivo* 2-photon microscopy, IOS and bulk loading of calcium indicator dyes, contradict these findings. Within the olfactory bulb glomerulus, odor stimulation resulted in a local increase in CBF which was strongly correlated, both spatially and temporally, with an increase in astrocytic [Ca^2+^]_i_ (Petzold et al., 2008). More recently, Lind et al. (2013) used signal-enhancing analysis of Ca^2+^ activity to give higher sensitivity to fast Ca^2+^ signals. This study demonstrates that, in contrast to the small proportion of astrocytes previously reported to exhibit fast [Ca^2+^]_i_ transients (Winship et al., 2007), in the whisker barrel cortex of adult mice 66% of astrocyte somata and 70% of processes exhibit a stimulus-evoked [Ca^2+^]_i_ elevation with rapid onset (peak ~100 ms) and short duration which precedes local vasodilations (Lind et al., 2013). While stimulus-evoked [Ca^2+^]_i_ transients occurring concurrently in neurons and astrocytes correlated with synaptic activity, only the astrocytic signals correlated with hemodynamic changes. Astrocytic calcium transients consisted of a fast response and, in ~10% of astrocytes, slow augmentation. The authors suggest that it is this slow component that has been previously reported by other studies and that it is their improved analysis method which enables the fast component to be detected.

## Are subcellular Ca^2+^ transients important?

In brain slices, it has been shown that calcium signals can occur in astrocytic processes in the absence of changes in the cell body (Di Castro et al., 2011). It may be that subcellular astrocyte calcium transients, e.g., those in the endfeet rather than those in the soma, are important for the regulation of CBF (McCaslin et al., 2011; Dunn et al., 2013; Lind et al., 2013). Devor's group reported that the onset of [Ca^2+^]_i_ transients in endfeet (which may precede those in the soma: Wang et al., 2006) were delayed relative to the onset of arteriole dilation at the same cortical depth (Nizar et al., 2013). However, Lind et al. (2013) demonstrated fast [Ca^2+^]_i_ transients within endfeet which preceeded local vasodilation. In order to investigate [Ca^2+^]_i_ transients in the astrocytic soma and/or processes, these studies, along with those of other groups (e.g., Dunn et al., 2013), utilized bulk loading of calcium indicator dye which lacks cellular specificity. The development of targeted expression of genetically induced calcium indicators will allow better dye localization and may result in the reliable detection of fast subcellular [Ca^2+^]_i_ transients. Such subcellular transients could result in the release of vasoactive substances, hence playing a role in the regulation of CBF. Although this technique has yet to reveal results *in vivo*, membrane-bound genetic calcium indicators have been shown to detect local, subcellular, calcium rises in cultured astrocytes (Shigetomi et al., 2010a,b).

Finally, the majority of published neurovascular coupling studies have been performed in the cortex of anesthetized animals. Anesthetics may disrupt important features of neurovascular coupling, thus acting as a confound in understanding the cellular mechanisms underlying the regulation of CBF in response to neural activity (Martin et al., 2012). Three commonly used anesthetic combinations (ketamine/xylazine, isoflurane, and urethane) have been found to significantly suppress sensory-evoked astrocyte [Ca^2+^]_i_ transients in mice (Thrane et al., 2012). Sensory-evoked [Ca^2+^]_i_ transients were found to be more delayed with a slower rise time and longer duration in anesthetized animals compared to awake animals (Thrane et al., 2012). Further studies in awake rodents, such as those performed by Martin et al. (2012), are required in order to fully investigate the role of astrocytes, and their sensory-evoked [Ca^2+^]_i_ transients, in neurovascular coupling.

## Conclusions

The work outlined here demonstrates that astrocytes are capable of eliciting both vasoconstriction and vasodilation of brain arterioles. A popular hypothesis of astrocytic control of CBF in response to neural activity has been that neuronally released glutamate acts on astrocytic mGluRs to raise astrocytic [Ca^2+^]_i_, initiating downstream production of AA and the formation and release of vasoactive substances (Zonta et al., 2003; Mulligan and MacVicar, 2004; Takano et al., 2006; Petzold et al., 2008). However, recent studies have called into question the role of mGluR5 and IP_3_-mediated downstream pathways in the functional hyperemia response (Gurden et al., 2006; Calcinaghi et al., 2011; Nizar et al., 2013; Sun et al., 2013). Evidence from the retina suggests that neuron-glia signaling may be mediated by neuronally released ATP acting on glial P2Y receptors rather than via activation of mGluRs by glutamate (Newman, 2005; Metea and Newman, 2006). Indeed, it has been shown that astrocyte [Ca^2+^]_i_ signals can be evoked by ATP in the cerebral cortex (Sun et al., 2013) and in cerebellar slices (Piet and Jahr, 2007; Habbas et al., 2011). Alternative hypotheses of astrocyte control of vessel diameter also include the efflux of K^+^ through Ca^2+^-activated K^+^ channels in astrocyte endfeet (Filosa et al., 2006), although the functional, *in vivo*, significance of this pathway remains to be demonstrated. The role of astrocyte [Ca^2+^]_i_ transients in the control of CBF *in vivo* during functional hyperemia remains controversial. An inability to observe Ca^2+^ transients that are fast enough for neurovascular coupling has called into question the impact of astrocytes on CBF regulation in response to neural activity (Nizar et al., 2013). Recent advances in data analysis techniques resulting in a higher sensitivity to fast Ca^2+^ signals may have overcome this problem (Lind et al., 2013), providing direct evidence for the existence of Ca^2+^ responses which are rapid enough to contribute to neurovascular coupling. It is, however, worth considering that while we study Ca^2+^ because we can currently visualize it, Ca^2+^-independent mechanisms such as those involving glutamate transport (Gurden et al., 2006; Petzold et al., 2008; Schummers et al., 2008) may play an important role in astrocyte-mediated regulation of CBF. A role for astrocytes in the control of CBF in pathology also remains a possibility (Chuquet et al., 2007). While the evidence suggests that astrocytes are important players in neurovascular coupling and functional hyperemia, the questions of whether astrocytes play a dominant role in triggering fast hemodynamic responses and, in particular, under what circumstances astrocytic Ca^2+^-mediated pathways are responsible, remain open. The exact mechanisms by which astrocytes are able to sense changes in neuronal activity and trigger the intracellular events regulating the resulting vascular response which underlies the fMRI BOLD signal remain unclear. Indeed, which pathway predominates may often result from the experimental model used. Other issues which remain to be solved are: what is the functional significance of astrocytic [Ca^2+^]_i_ transients in awake animals? Under what circumstances are mGluR-mediated vasodilation and constriction important? What are the messengers underlying neurovascular coupling in healthy and diseased brain? Do slow astrocyte [Ca^2+^]_i_ signals contribute to the sustained hemodynamic response? Research on this topic must continue. New technologies such as targeted genetic encoding of calcium indicators, optogenetics, and transgenic mouse lines allowing astrocyte physiology specifically to be altered will help us move forward with this research. Only by fully understanding the cellular mechanisms underlying functional hyperemia and the resulting BOLD signal will we be able to accurately interpret the BOLD fMRI signal in health and disease.

### Conflict of interest statement

The author declares that the research was conducted in the absence of any commercial or financial relationships that could be construed as a potential conflict of interest.

## References

[B1] Akgoren N. Fabricius M. Lauritzen M. (1994). Importance of nitric oxide for local increases of blood flow in rat cerebellar cortex during electrical stimulation. Proc. Natl. Acad. Sci. U.S.A. 91, 5903–5907 10.1073/pnas.91.13.5903 7517038 PMC44105

[B2] Akgoren N. Mathiesen C. Rubin I. Lauritzen M. (1997). Laminar analysis of activity-dependent increases of CBF in rat cerebellar cortex: dependence on synaptic strength. Am. J. Physiol. 273, H1166–H1176 9321803 10.1152/ajpheart.1997.273.3.H1166

[B3] Aronica E. Van Vliet E. A. Mayboroda O. A. Troost D. Da Silva F. H. Gorter J. A. (2000). Upregulation of metabotropic glutamate receptor subtype mGluR3 and mGluR5 in reactive astrocytes in a rat model of mesial temporal lobe epilepsy. Eur. J. Neurosci. 12, 2333–2344 10.1046/j.1460-9568.2000.00131.x 10947812

[B4] Attwell D. Buchan A. M. Charpak S. Lauritzen M. MacVicar B. A. Newman E. A. (2010). Glial and neuronal control of brain blood flow. Nature 468, 232–243 10.1038/nature09613 21068832 PMC3206737

[B5] Blanco V. M. Stern J. E. Filosa J. A. (2008). Tone-dependent vascular responses to astrocyte-derived signals. Am. J. Physiol. Heart Circ. Physiol. 294, H2855–H2863 10.1152/ajpheart.91451.2007 18456724 PMC3962545

[B6] Blum A. E. Joseph S. M. Przybylski R. J. Dubyak G. R. (2008). Rho-family GTPases modulate Ca(2+)-dependent ATP release from astrocytes. Am. J. Physiol. Cell Physiol. 295, C231–C241 10.1152/ajpcell.00175.2008 18495810 PMC2493544

[B7] Cahoy J. D. Emery B. Kaushal A. Foo L. C. Zamanian J. L. Christopherson K. S. (2008). A transcriptome database for astrocytes, neurons, and oligodendrocytes: a new resource for understanding brain development and function. J. Neurosci. 28, 264–278 10.1523/JNEUROSCI.4178-07.2008 18171944 PMC6671143

[B8] Calcinaghi N. Jolivet R. Wyss M. T. Ametamey S. M. Gasparini F. Buck A. (2011). Metabotropic glutamate receptor mGluR5 is not involved in the early hemodynamic response. J. Cereb. Blood Flow Metab. 31, e1–e10 10.1038/jcbfm.2011.96 21731033 PMC3185891

[B9] Carrasquero L. M. Delicado E. G. Bustillo D. Gutierrez-Martin Y. Artalejo A. R. Miras-Portugal M. T. (2009). P2X7 and P2Y13 purinergic receptors mediate intracellular calcium responses to BzATP in rat cerebellar astrocytes. J. Neurochem. 110, 879–889 10.1111/j.1471-4159.2009.06179.x 19457067

[B10] Cauli B. Hamel E. (2010). Revisiting the role of neurons in neurovascular coupling. Front. Neuroenergetics 2:9 10.3389/fnene.2010.00009 20616884 PMC2899521

[B11] Chan B. S. Endo S. Kanai N. Schuster V. L. (2002). Identification of lactate as a driving force for prostanoid transport by prostaglandin transporter PGT. Am. J. Physiol. Renal Physiol. 282, F1097–F1102 10.1152/ajprenal.00151.2001 11997326

[B12] Chuquet J. Hollender L. Nimchinsky E. A. (2007). High-resolution *in vivo* imaging of the neurovascular unit during spreading depression. J. Neurosci. 27, 4036–4044 10.1523/JNEUROSCI.0721-07.2007 17428981 PMC6672520

[B13] Dabertrand F. Hannah R. M. Pearson J. M. Hill-Eubanks D. C. Brayden J. E. Nelson M. T. (2013). Prostaglandin E2, a postulated astrocyte-derived neurovascular coupling agent, constricts rather than dilates parenchymal arterioles. J. Cereb. Blood Flow Metab. 33, 479–482 10.1038/jcbfm.2013.9 23385200 PMC3618402

[B14] Devor A. Dunn A. K. Andermann M. L. Ulbert I. Boas D. A. Dale A. M. (2003). Coupling of total hemoglobin concentration, oxygenation, and neural activity in rat somatosensory cortex. Neuron 39, 353–359 10.1016/S0896-6273(03)00403-3 12873390

[B15] Di Castro M. A. Chuquet J. Liaudet N. Bhaukaurally K. Santello M. Bouvier D. (2011). Local Ca^2+^ detection and modulation of synaptic release by astrocytes. Nat. Neurosci. 14, 1276–1284 10.1038/nn.2929 21909085

[B16] Dirnagl U. Niwa K. Lindauer U. Villringer A. (1994). Coupling of cerebral blood flow to neuronal activation: role of adenosine and nitric oxide. Am. J. Physiol. 267, H296–H301 8048594 10.1152/ajpheart.1994.267.1.H296

[B17] Dunn K. M. Hill-Eubanks D. C. Liedtke W. B. Nelson M. T. (2013). TRPV4 channels stimulate Ca^2+^-induced Ca^2+^ release in astrocytic endfeet and amplify neurovascular coupling responses. Proc. Natl. Acad. Sci. U.S.A. 110, 6157–6162 10.1073/pnas.1216514110 23530219 PMC3625327

[B18] Farooqui A. A. Yang H. C. Rosenberger T. A. Horrocks L. A. (1997). Phospholipase A2 and its role in brain tissue. J. Neurochem. 69, 889–901 10.1046/j.1471-4159.1997.69030889.x 9282910

[B19] Fergus A. Lee K. S. (1997). Regulation of cerebral microvessels by glutamatergic mechanisms. Brain Res. 754, 35–45 10.1016/S0006-8993(97)00040-1 9134957

[B20] Filosa J. A. Bonev A. D. Nelson M. T. (2004). Calcium dynamics in cortical astrocytes and arterioles during neurovascular coupling. Circ. Res. 95, e73–e81 10.1161/01.RES.0000148636.60732.2e 15499024

[B21] Filosa J. A. Bonev A. D. Straub S. V. Meredith A. L. Wilkerson M. K. Aldrich R. W. (2006). Local potassium signaling couples neuronal activity to vasodilation in the brain. Nat. Neurosci. 9, 1397–1403 10.1038/nn1779 17013381

[B22] Fleming I. (2001). Cytochrome p450 and vascular homeostasis. Circ. Res. 89, 753–762 10.1161/hh2101.099268 11679404

[B23] Funk R. H. (1997). Blood supply of the retina. Ophthalmic Res. 29, 320–325 10.1159/000268030 9323723

[B24] Gerrits R. J. Stein E. A. Greene A. S. (2002). Ca(2++-activated potassium (K(Ca)) channel inhibition decreases neuronal activity-blood flow coupling. Brain Res. 948, 108–116 10.1016/S0006-8993(02)02957-8 12383961

[B25] Gordon G. R. Choi H. B. Rungta R. L. Ellis-Davies G. C. MacVicar B. A. (2008). Brain metabolism dictates the polarity of astrocyte control over arterioles. Nature 456, 745–749 10.1038/nature07525 18971930 PMC4097022

[B26] Gurden H. Uchida N. Mainen Z. F. (2006). Sensory-evoked intrinsic optical signals in the olfactory bulb are coupled to glutamate release and uptake. Neuron 52, 335–345 10.1016/j.neuron.2006.07.022 17046695

[B27] Habbas S. Ango F. Daniel H. Galante M. (2011). Purinergic signalling in teh cerebellum: Bergmann glial cells express functional ionotropic P2X7 receptors. Glia 59, 1800–1812 10.1002/glia.21224 21830236

[B28] Hu Y. Wilson G. S. (1997). A temporary local energy pool coupled to neuronal activity: fluctuations of extracellular lactate levels in rat brain monitored with rapid-response enzyme-based sensor. J. Neurochem. 69, 1484–1490 10.1046/j.1471-4159.1997.69041484.x 9326277

[B29] Iadecola C. Nedergaard M. (2007). Glial regulation of the cerebral microvasculature. Nat. Neurosci. 10, 1369–1376 10.1038/nn2003 17965657

[B30] Kleinfeld D. Mitra P. P. Helmchen F. Denk W. (1998). Fluctuations and stimulus-induced changes in blood flow observed in individual capillaries in layers 2 through 4 of rat neocortex. Proc. Natl. Acad. Sci. U.S.A. 95, 15741–15746 10.1073/pnas.95.26.15741 9861040 PMC28114

[B31] Kuffler S. W. Nicholls J. G. Orkand R. K. (1966). Physiological properties of glial cells in the central nervous system of amphibia. J. Neurophysiol. 29, 768–787 5966434 10.1152/jn.1966.29.4.768

[B32] Kuschinsky W. Wahl M. (1978). Local chemical and neurogenic regulation of cerebral vascular resistance. Physiol. Rev. 58, 656–689 28540 10.1152/physrev.1978.58.3.656

[B32a] Lange A. Gebremedhin D. Narayanan J. Harder D. (1997). 20-Hydroxyeicosatetraenoic acid-induced vasoconstriction and inhibition of potassium current in cerebral vascular smooth muscle is dependent on activation of protein kinase C. J. Biol. Chem. 272, 27345–27352 10.1074/jbc.272.43.27345 9341185

[B33] Lecrux C. Toussay X. Kocharyan A. Fernandes P. Neupane S. Levesque M. (2011). Pyramidal neurons are “neurogenic hubs” in the neurovascular coupling response to whisker stimulation. J. Neurosci. 31, 9836–9847 10.1523/JNEUROSCI.4943-10.2011 21734275 PMC6703330

[B34] Leithner C. Royl G. Offenhauser N. Fuchtemeier M. Kohl-Bareis M. Villringer A. (2010). Pharmacological uncoupling of activation induced increases in CBF and CMRO2. J. Cereb. Blood Flow Metab. 30, 311–322 10.1038/jcbfm.2009.211 19794398 PMC2949119

[B35] Lin A. L. Fox P. T. Hardies J. Duong T. Q. Gao J. H. (2010). Nonlinear coupling between cerebral blood flow, oxygen consumption, and ATP production in human visual cortex. Proc. Natl. Acad. Sci. U.S.A. 107, 8446–8451 10.1073/pnas.0909711107 20404151 PMC2889577

[B36] Lind B. L. Brazhe A. R. Jessen S. B. Tan F. C. Lauritzen M. J. (2013). Rapid stimulus-evoked astrocyte Ca^2+^ elevations and hemodynamic responses in mouse somatosensory cortex *in vivo*. Proc. Natl. Acad. Sci. U.S.A. 110, E4678–E4687 10.1073/pnas.1310065110 24218625 PMC3845114

[B37] Lindauer U. Leithner C. Kaasch H. Rohrer B. Foddis M. Fuchtemeier M. (2010). Neurovascular coupling in rat brain operates independent of hemoglobin deoxygenation. J. Cereb. Blood Flow Metab. 30, 757–768 10.1038/jcbfm.2009.259 20040927 PMC2949158

[B38] Liu X. Li C. Falck J. R. Harder D. R. Koehler R. C. (2012). Relative contribution of cyclooxygenases, epoxyeicosatrienoic acids, and pH to the cerebral blood flow response to vibrissal stimulation. Am. J. Physiol. Heart. Circ. Physiol. 302, H1075–H1085 10.1152/ajpheart.00794.2011 22198176 PMC3311453

[B39] Martin C. Zheng Y. Sibson N. R. Mayhew J. E. Berwick J. (2012). Complex spatiotemporal haemodynamic response following sensory stimulation in the awake rat. Neuroimage 66C, 1–8 10.1016/j.neuroimage.2012.10.006 23063446 PMC3556776

[B40] McCalden T. A. Nath R. G. Thiele K. (1984). The role of prostacyclin in the hypercapnic and hypoxic cerebrovascular dilations. Life Sci. 34, 1801–1807 10.1016/0024-3205(84)90672-6 6376989

[B41] McCaslin A. F. Chen B. R. Radosevich A. J. Cauli B. Hillman E. M. (2011). *In vivo* 3D morphology of astrocyte-vasculature interactions in the somatosensory cortex: implications for neurovascular coupling. J. Cereb. Blood Flow Metab. 31, 795–806 10.1038/jcbfm.2010.204 21139630 PMC3063633

[B42] Metea M. R. Kofuji P. Newman E. A. (2007). Neurovascular coupling is not mediated by potassium siphoning from glial cells. J. Neurosci. 27, 2468–2471 10.1523/JNEUROSCI.3204-06.2007 17344384 PMC2289782

[B43] Metea M. R. Newman E. A. (2006). Glial cells dilate and constrict blood vessels: a mechanism of neurovascular coupling. J. Neurosci. 26, 2862–2870 10.1523/JNEUROSCI.4048-05.2006 16540563 PMC2270788

[B44] Mishra A. Hamid A. Newman E. A. (2011). Oxygen modulation of neurovascular coupling in the retina. Proc. Natl. Acad. Sci. U.S.A. 108, 17827–17831 10.1073/pnas.1110533108 22006332 PMC3203806

[B45] Mulligan S. J. MacVicar B. A. (2004). Calcium transients in astrocyte endfeet cause cerebrovascular constrictions. Nature 431, 195–199 10.1038/nature02827 15356633

[B46] Murphy K. Gerzanich V. Zhou H. Ivanova S. Dong Y. Hoffman G. (2003). Adenosine-A2a receptor down-regulates cerebral smooth muscle L-type Ca^2+^ channel activity via protein tyrosine phosphatase, not cAMP-dependent protein kinase. Mol. Pharmacol. 64, 640–649 10.1124/mol.64.3.640 12920200

[B47] Newman E. A. (2005). Calcium increases in retinal glial cells evoked by light-induced neuronal activity. J. Neurosci. 25, 5502–5510 10.1523/JNEUROSCI.1354-05.2005 15944378 PMC1405916

[B48] Niwa K. Araki E. Morham S. G. Ross M. E. Iadecola C. (2000). Cyclooxygenase-2 contributes to functional hyperemia in whisker-barrel cortex. J. Neurosci. 20, 763–770 10632605 10.1523/JNEUROSCI.20-02-00763.2000 PMC6772412

[B49] Niwa K. Haensel C. Ross M. E. Iadecola C. (2001). Cyclooxygenase-1 participates in selected vasodilator responses of the cerebral circulation. Circ. Res. 88, 600–608 10.1161/01.RES.88.6.600 11282894

[B50] Nizar K. Uhlirova H. Tian P. Saisan P. A. Cheng Q. Reznichenko L. (2013). *In vivo* stimulus-induced vasodilation occurs without IP3 receptor activation and may precede astrocytic calcium increase. J. Neurosci. 33, 8411–8422 10.1523/JNEUROSCI.3285-12.2013 23658179 PMC3712855

[B51] Offenhauser N. Thomsen K. Caesar K. Lauritzen M. (2005). Activity-induced tissue oxygenation changes in rat cerebellar cortex: interplay of postsynaptic activation and blood flow. J. Physiol. 565, 279–294 10.1113/jphysiol.2005.082776 15774524 PMC1464487

[B52] Pangrsic T. Potokar M. Stenovec M. Kreft M. Fabbretti E. Nistri A. (2007). Exocytotic release of ATP from cultured astrocytes. J. Biol. Chem. 282, 28749–28758 10.1074/jbc.M700290200 17627942

[B53] Paulson O. B. Newman E. A. (1987). Does the release of potassium from astrocyte endfeet regulate cerebral blood flow? Science 237, 896–898 10.1126/science.3616619 3616619 PMC2505270

[B54] Petzold G. C. Albeanu D. F. Sato T. F. Murthy V. N. (2008). Coupling of neural activity to blood flow in olfactory glomeruli is mediated by astrocytic pathways. Neuron 58, 897–910 10.1016/j.neuron.2008.04.029 18579080 PMC2922004

[B55] Petzold G. C. Murthy V. N. (2011). Role of astrocytes in neurovascular coupling. Neuron 71, 782–797 10.1016/j.neuron.2011.08.009 21903073

[B56] Piet R. Jahr C. E. (2007). Glutamatergic and purinergic receptor-mediated calcium transients in Bergmann glial cells. J. Neurosci. 27, 4027–4035 10.1523/JNEUROSCI.0462-07.2007 17428980 PMC2671228

[B57] Roman R. J. (2002). P-450 metabolites of arachidonic acid in the control of cardiovascular function. Physiol. Rev. 82, 131–185 10.1152/physrev.00021.2001 11773611

[B58] Roy C. S. Sherrington C. S. (1890). On the regulation of the blood-supply of the brain. J. Physiol. 11, 85–158 16991945 10.1113/jphysiol.1890.sp000321 PMC1514242

[B59] Schulz K. Stydekum E. Krueppel R. Engelbrecht C. J. Schlegel F. Schroter A. (2012). Simultaneous BOLD fMRI and fiber-optic calcium recording in rat neocortex. Nat. Methods 9, 597–602 10.1038/nmeth.2013 22561989

[B60] Schummers J. Yu H. Sur M. (2008). Tuned responses of astrocytes and their influence on hemodynamic signals in the visual cortex. Science 320, 1638–1643 10.1126/science.1156120 18566287

[B61] Shi Y. Liu X. Gebremedhin D. Falck J. R. Harder D. R. Koehler R. C. (2008). Interaction of mechanisms involving epoxyeicosatrienoic acids, adenosine receptors, and metabotropic glutamate receptors in neurovascular coupling in rat whisker barrel cortex. J. Cereb. Blood Flow Metab. 28, 111–125 10.1038/sj.jcbfm.9600511 17519974 PMC2204069

[B62] Shigetomi E. Kracun S. Khakh B. S. (2010a). Monitoring astrocyte calcium microdomains with improved membrane targeted GCaMP reporters. Neuron Glia Biol. 6, 183–191 10.1017/S1740925X10000219 21205365 PMC3136572

[B63] Shigetomi E. Kracun S. Sofroniew M. V. Khakh B. S. (2010b). A genetically targeted optical sensor to monitor calcium signals in astrocyte processes. Nat. Neurosci. 13, 759–766 10.1038/nn.2557 20495558 PMC2920135

[B64] Simard M. Arcuino G. Takano T. Liu Q. S. Nedergaard M. (2003). Signaling at the gliovascular interface. J. Neurosci. 23, 9254–9262 14534260 10.1523/JNEUROSCI.23-27-09254.2003 PMC6740832

[B65] Sloan H. L. Austin V. C. Blamire A. M. Schnupp J. W. Lowe A. S. Allers K. A. (2010). Regional differences in neurovascular coupling in rat brain as determined by fMRI and electrophysiology. Neuroimage 53, 399–411 10.1016/j.neuroimage.2010.07.014 20633665

[B66] Sun W. McConnell E. Pare J. F. Xu Q. Chen M. Peng W. (2013). Glutamate-dependent neuroglial calcium signaling differs between young and adult brain. Science 339, 197–200 10.1126/science.1226740 23307741 PMC3569008

[B67] Takano T. Tian G. F. Peng W. Lou N. Libionka W. Han X. (2006). Astrocyte-mediated control of cerebral blood flow. Nat. Neurosci. 9, 260–267 10.1038/nn1623 16388306

[B68] Thrane A. S. Rangroo Thrane V. Zeppenfeld D. Lou N. Xu Q. Nagelhus E. A. (2012). General anesthesia selectively disrupts astrocyte calcium signaling in the awake mouse cortex. Proc. Natl. Acad. Sci. U.S.A. 109, 18974–18979 10.1073/pnas.1209448109 23112168 PMC3503159

[B69] Wang F. Smith N. A. Xu Q. Goldman S. Peng W. Huang J. H. (2013). Photolysis of caged Ca^2+^ but not receptor-mediated Ca^2+^ signaling triggers astrocytic glutamate release. J. Neurosci. 33, 17404–17412 10.1523/JNEUROSCI.2178-13.2013 24174673 PMC3812507

[B70] Wang X. Lou N. Xu Q. Tian G. F. Peng W. G. Han X. (2006). Astrocytic Ca^2+^ signaling evoked by sensory stimulation *in vivo*. Nat. Neurosci. 9, 816–823 10.1038/nn1703 16699507

[B71] Winship I. R. Plaa N. Murphy T. H. (2007). Rapid astrocyte calcium signals correlate with neuronal activity and onset of the hemodynamic response *in vivo*. J. Neurosci. 27, 6268–6272 10.1523/JNEUROSCI.4801-06.2007 17554000 PMC6672142

[B72] Xu H. L. Pelligrino D. A. (2007). ATP release and hydrolysis contribute to rat pial arteriolar dilatation elicited by neuronal activation. Exp. Physiol. 92, 647–651 10.1113/expphysiol.2006.036863 17468204

[B73] Zonta M. Angulo M. C. Gobbo S. Rosengarten B. Hossmann K. A. Pozzan T. (2003). Neuron-to-astrocyte signaling is central to the dynamic control of brain microcirculation. Nat. Neurosci. 6, 43–50 10.1038/nn980 12469126

